# Public health round-up

**DOI:** 10.2471/BLT.21.010821

**Published:** 2021-08-01

**Latest Ebola outbreak ends**

A health worker takes the temperature of a woman passing through a checkpoint in Gouéké in Guinea where a cluster of Ebola virus disease cases was reported in February 2021. The government declared the outbreak to be over on 19 June.

**Figure:**
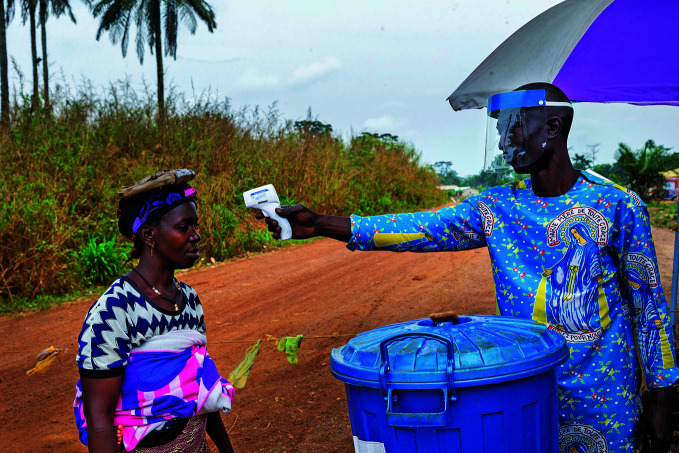
WHO/Ahmed Jallanzo

## Childhood vaccine shortfall

An estimated 23 million children missed out on basic childhood vaccines in 2020, the highest number since 2009 and 3.7 million more than in 2019. This is according to data published by the World Health Organization (WHO) and the United Nations International Children’s Emergency Fund (UNICEF) on 15 July.

The first official figures to reflect global service disruptions due to coronavirus disease 2019 (COVID-19), the data show that most countries experienced drops in childhood vaccination rates. Concerningly, up to 17 million children did not receive a single vaccine during 2020, most of them living in in communities affected by conflict, in underserved remote places, or in informal developments where they face multiple deprivations including limited access to basic health and key social services.

“Multiple disease outbreaks would be catastrophic for communities and health systems already battling COVID-19, making it more urgent than ever to invest in childhood vaccination and ensure every child is reached,” said WHO Director-General Tedros Adhanom Ghebreyesus.

https://bit.ly/3xLePLx

## New medicines to treat severe COVID-19

WHO recommended the use of interleukin-6 receptor blockers in the treatment of patients severely or critically ill with COVID-19.

Announced on 6 July, the recommendation is based on an analysis of data from over 10 000 patients enrolled in 27 clinical trials which showed the medicines to be lifesaving, especially when administered alongside corticosteroids. The medicines are the first to be found effective against COVID-19 since corticosteroids were recommended by WHO in September 2020.

WHO called on manufacturers to reduce prices of the medicines, make supplies available to low- and middle-income countries, especially where COVID-19 cases are surging, and agree to voluntary licensing agreements.

https://bit.ly/3kdI8Cv

## COVID-19 technology transfer hub

WHO and its COVAX partners are working with a South African consortium to establish the country’s first technology transfer hub for the manufacture of messenger ribonucleic acid (mRNA) vaccine against COVID-19.

Announced on 21 June, the initiative follows WHO’s call to establish such hubs to boost production of and access to COVID vaccines. As of 21 June, partners were negotiating details with the Government of South Africa and public and private partners inside the country and from around the world.

South African President Cyril Ramaphosa said: “South Africa welcomes the opportunity to host a vaccine technology transfer hub and to build on the capacity and expertise that already exists on the continent.”

https://bit.ly/3hZs7xi

## Guinea Ebola outbreak ends

The Ebola outbreak that began in Guinea on 14 February 2021 was declared to have ended on 19 June by the ministry of health in that country. Sixteen people were infected during the outbreak, 12 of whom died.

Shortly after the infections were detected, national health authorities, with support from WHO and partners, mounted a swift response, tapping into the expertise gained in fighting recent outbreaks both in Guinea and in the Democratic Republic of the Congo.

WHO helped ship around 24 000 Ebola vaccine doses and supported the vaccination of nearly 11 000 people at high risk, including over 2800 frontline workers. More than 100 WHO experts were on the ground coordinating key aspects of the response such as infection prevention and control, disease surveillance, testing, vaccination and treatment using new drugs. Collaboration with communities was also enhanced to raise awareness about the virus and ensure community involvement in and ownership of efforts to curb the disease.

https://bit.ly/3i4ZGhB

## World hunger worsens

There was a dramatic worsening of world hunger in 2020, much of it likely related to COVID-19 repercussions. This is according to *The state of food security and nutrition in the world* which was jointly published by the Food and Agriculture Organization (FAO) of the United Nations (UN), the International Fund for Agricultural Development (IFAD), UNICEF, the UN World Food Programme (WFP) and WHO on 12 July.

The first global assessment of its kind in the pandemic era, the report estimates that around a tenth of the global population – up to 811 million people – were undernourished last year, a number that raises concerns regarding sustainable development goal 2 which is to end hunger by 2030.

https://bit.ly/2UEQys2

## China malaria free

China has been awarded a malaria-free certification by WHO. Announced on 30 June, the certification adds China to the growing number of countries demonstrating that a malaria-free future is a viable goal.

China applied for an official WHO certification of malaria elimination in 2020 after reporting 4 consecutive years of zero indigenous cases. Members of the independent Malaria Elimination Certification Panel travelled to China in May 2021 to verify the country’s malaria-free status as well as its programme to prevent re-establishment of the disease.

China is the first country in the WHO Western Pacific Region to be awarded a malaria-free certification in more than 3 decades. Other countries in the region that have achieved this status include Australia (1981), Singapore (1982) and Brunei Darussalam (1987).

https://bit.ly/2UEWbqb

## Human genome editing guidance

WHO released two reports providing the first global guidance on the application of human genome editing as a tool for public health, with an emphasis on safety, effectiveness and ethical use.

Produced by the WHO-established Expert Advisory Committee on Developing Global Standards for Governance and Oversight of Human Genome Editing, the reports are the fruit of the first broad, global consultation looking at somatic, germline and heritable human genome editing.

The reports include recommendations on the governance and oversight of human genome editing in nine key areas and focus on systems-level improvements needed to build capacity in all countries to ensure that human genome editing is used safely, effectively and ethically.

Hailing the reports as a leap forward for this rapidly emerging field, WHO’s Chief Scientist, Dr Soumya Swaminathan, said, “As global research delves deeper into the human genome, we must minimize risks and leverage ways that science can drive better health for everyone, everywhere.”

https://bit.ly/3wxzTnd

## Artificial intelligence for health

WHO published its first global report on artificial intelligence (AI) for health. Released on 28 June, *Ethics and governance of artificial intelligence for health* is the result of 2 years of consultations by a panel of WHO-appointed experts and stresses the need to embed considerations of ethics and human rights in the design, deployment and use of AI technologies and systems.

“This important new report provides a valuable guide for countries on how to maximize the benefits of AI, while minimizing its risks and avoiding its pitfalls,” said WHO Director-General Tedros Adhanom Ghebreyesus.

https://bit.ly/3AUB0AT

**Cover photo**

Arrival of vaccines at the Central Vaccine Store in Nepal, which received nearly 2 million doses of the Johnson & Johnson COVID-19 vaccine through the COVAX Facility’s dose-sharing scheme.

**Figure:**
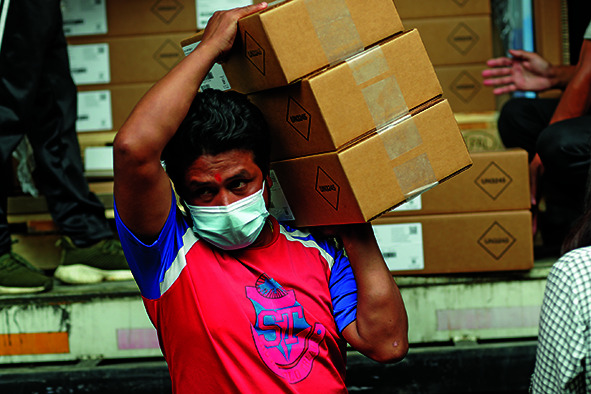
UNICEF/Laxmi Prasad Ngakhusi

## New cervical screening guidelines

WHO published new guidance for screening and treatment to prevent cervical cancer. Released on 6 July, the guidance recommends the use of a human papillomavirus (HPV) DNA-based test as the preferred method of screening, rather than visual inspection with acetic acid or cytology commonly known as a ‘Pap smear’, which is currently the most commonly used method to detect pre-cancer lesions.

HPV-DNA testing detects high-risk strains of HPV which cause almost all cervical cancers and, unlike tests that rely on visual inspection, is an objective diagnostic leaving no space for misinterpretation of results.

https://bit.ly/2U3Wc71

## Cataloguing tuberculosis mutations and drug resistance

The first WHO catalogue of mutations in the *Mycobacterium tuberculosis* genome complex and their association with drug resistance was released on 25 June. The catalogue provides a standard reference for the interpretation of mutations conferring resistance to all first-line and a variety of second-line tuberculosis drugs.

Summarizing the analysis of over 38 000 isolates with matched data on whole genome sequencing and phenotypic drug susceptibility testing from over 40 countries on 13 anti-tuberculosis medicines, the catalogue lists over 17 000 mutations, their frequency and association with drug resistance.

https://bit.ly/3hxn1JC

**Looking ahead**

1–7 August. World Breastfeeding Week. https://bit.ly/3w8feWC

7–9 September. 5th international UV & skin cancer prevention conference. https://bit.ly/3qF7xWT

14–30 September. United Nations Food Systems Summit. https://bit.ly/3A8zYRD

